# Acyl-CoA-binding protein family members in laticifers are possibly involved in lipid and latex metabolism of *Hevea brasiliensis* (the Para rubber tree)

**DOI:** 10.1186/s12864-017-4419-6

**Published:** 2018-01-02

**Authors:** Zhiyi Nie, Yihang Wang, Chuntai Wu, Yu Li, Guijuan Kang, Huaide Qin, Rizhong Zeng

**Affiliations:** 1 Rubber Research Institute & Key Laboratory of Biology and Genetic Resources of Rubber Trees, Ministry of Agriculture of China, Chinese Academy of Tropical Agricultural Sciences, Danzhou, Hainan 571737 China; 2 College of Agriculture, Hainan University, Haikou, 570228 China

**Keywords:** *Hevea brasiliensis*, Acyl-CoA-binding protein (ACBP), Laticifer, Latex production, Rubber biosynthesis

## Abstract

**Background:**

Acyl-CoA-binding proteins (ACBPs) are mainly involved in acyl-CoA ester binding and trafficking in eukaryotic cells, and their various functions have been characterized in model plants, such as *Arabidopsis thaliana* (*A. thaliana*), *Oryza sativa* (rice), and other plant species. In the present study, genome-wide mining and expression analysis of ACBP genes was performed on *Hevea brasiliensis* (the para rubber tree), the most important latex-producing crop in the world.

**Results:**

Six members of the *H. brasiliensis* ACBP family genes, designated *HbACBP1*-*HbACBP6*, were identified from the *H. brasiliensis* genome. They can be categorized into four classes with different amino acid sequences and domain structures based on the categorization of their *A. thaliana* counterparts. Phylogenetic analysis shows that the HbACBPs were clustered with those of other closely related species, such as *Manihot esculenta*, *Ricinus communis*, and *Jatropha carcas*, but were further from those of *A. thaliana*, a distantly related species. Expression analysis demonstrated that the HbACBP1 and HbACBP2 genes are more prominently expressed in *H. brasiliensis* latex, and their expression can be significantly enhanced by bark tapping (a mechanical wound) and jasmonic acid stimulation, whereas *HbACBP3*-*HbACBP6* had almost the same expression patterns with relatively high levels in mature leaves and male flowers, but a markedly low abundance in the latex. HbACBP1 and HbACBP2 may have crucial roles in lipid and latex metabolism in laticifers, so their subcellular location was further investigated and the results indicated that HbACBP1 is a cytosol protein, whereas HbACBP2 is an endoplasmic reticulum-associated ACBP.

**Conclusions:**

In this study, the *H. brasiliensis* ACBP family genes were identified. Phylogenetic analyses of the HbABCPs indicate that there is a high conservation and evolutionary relationship between ACBPs in land plants. The HbACBPs are organ/tissue-specifically expressed and have different expression patterns in response to stimulation by bark tapping or ethrel/jasmonic acid. HbACBP1 and HbACBP2 are two important latex ACBPs that might be involved in the lipid and latex metabolism. The results may provide valuable information for further investigations into the biological functions of HbACBPs during latex metabolism and stress responses in *H. brasiliensis*.

**Electronic supplementary material:**

The online version of this article (10.1186/s12864-017-4419-6) contains supplementary material, which is available to authorized users.

## Background

Acyl-CoA-binding proteins (ACBPs) have a conserved acyl-CoA-binding domain (ACBD) that binds to ligands of medium- and long-chain acyl-CoA esters with very high affinities [1–3]. ACBPs are mainly involved in intracellular transport and acyl-CoA pool formation during lipid metabolism [4, 5]. The comprehensively characterized ACBP contains only about 90 amino acids (with an approximate molecular weight of 10 kDa), which is highly conserved and found in all four eukaryotic kingdoms and in some eubacterial species [6]. Plant ACBPs have been investigated in the model plants *Arabidopsis thaliana* (*A. thaliana*) and *Oryza sativa* (rice) [7, 8], and more ACBP genes have recently been characterized after genome sequencing other plant species e.g. *Brassica napus* [9], and the tung tree (*Vernicia fordii*) [10]. Phylogenetic analysis of ACBPs extracted from 30 plant genomes demonstrated that plant ACBPs can be grouped into four classes that have clear differences in their amino acids and structural domains. Classes I and III possess only one ACBD, class II contains one ACBD plus ankyrin repeats, and class IV contains one ACBD plus kelch motifs [11].

The *A. thaliana* ACBP family has six members (designated AtACBP1-AtACBP6) that have diverse functions in a number of plant developmental processes and responses to biotic or abiotic stresses [11–13]. AtACBP1 and AtACBP2, belong to class II ACBPs with ankyrin repeats and contribute to heavy metal tolerance (such as Pb and Cd), possibly by maintaining or repairing the plasma membrane (PM) when they bind acyl-CoA esters [14–16]. AtACBP3 is an apoplast-targeted class III ACBP that is involved in leaf senescence and disease resistance [16–18]. Whereas AtACBP1-AtACBP3 are PM- or endoplasmic reticulum (ER)-associated ACBPs, the remaining AtACBP4-AtACBP6 are predominantly cytosolic [7, 19]. The cytosolic AtACBPs have multiple functions and are involved in seed development and oil synthesis through intracellular acyl-CoA trafficking and lipid metabolism [13, 20]. A recent study demonstrated that AtACBP6 is involved in jasmonic acid (JA) biosynthesis in *A. thaliana* phloem because it affects COMATOSE (CTS) function and regulates oxylipin levels [21]. This study implied the possible relationship between lipid metabolism and the JA signaling pathway used during the production of the secondary metabolites produced by several economic crops, including *Hevea brasiliensis* (*H. brasiliensis*, the Para rubber tree), which is the most important latex-producing species and is the primary source of high-quality natural rubber worldwide.

The intense metabolic activity of the laticifers is a prerequisite for the reconstitution of lost intracellular components in the exported latex (the laticifer’s cytoplasm) of rubber trees after regular bark tapping. The highly specialized laticifer system has no plasmodesmata and is therefore apoplastically isolated from the adjacent cells in the inner bark of rubber trees [22]. This means that laticifers have unique molecular characteristics, which possibly include lipid metabolism mediated by ACBPs like those of other plants. So far, *H.brasiliensis* ACBPs (HbACBPs) have not yet been identified. In this study, the genome-wide identification of the HbACBPs was performed by mining the recently published *H. brasiliensis* genome sequences [23]. The HbACBPs that were predominantly expressed in the laticifers were then investigated in detail. This study provides valuable information for further investigations into the biological functions of HbACBPs during latex metabolism and stress responses in rubber trees.

## Methods

### Plant materials and treatments

Rubber tree clones (Reyan 7–33-97) were planted at the experimental farm of the Chinese Academy of Tropical Agricultural Sciences (CATAS), Hainan, China. Those with similar stem girths were selected for the experiments, and the harvesting system was a half spiral with a tapping frequency of 3 days (S/2 d/3 system). The plant tissues, latex, male and female flowers, leaves and barks were collected from the rubber trees that had not been previously treated with anything, but were regularly tapped for 2 years, and these trees were also used for stimulation experiments with JA (Sigma-Aldrich, St. Louis, MO, USA) or ethrel/ethephon (ET) (Sigma-Aldrich, St. Louis, MO, USA) according to previously described methods [24]. For the bark tapping (mechanical wound) assays, 7-year-old mature and virgin rubber trees were sequentially tapped seven times using the S/2 d/3 system. In addition, cultivars of rubber tree (clones Reyan 7–33-97, Reyan 7–20-59, Reyan 8–79, PR107, RRIM600) and Amazon wild germplasms (MT/IT/13 29/8, MT/C/2 10/49, RO/PB/1 2/78, RO/C/9 23/219, AC/F/7 38/63) were used for comparison of the expression difference of *HbACBP1* and *HbACBP2* among different rubber tree clones/germplasms, and they are planted at the National Rubber Tree Germplasm Repository, Hainan, China. Fresh latex was collected from these rubber trees using a S/2 d/3 system.

### Sample collection, dry rubber yield measurement and total RNA preparation

All the samples were collected in a thermo bottle containing liquid nitrogen and then immediately stored at −80 °C. Each sample included three independent biological replicates, and each biological replicate comprised the samples collected from six trees (cultivars) or two trees (Amazon wild germplasms). The dry rubber yield of the rubber trees was measured as described by Tungngoen et al. [25]. Total RNAs were isolated using the universal plant total RNA extraction kit (BioTeke, Beijing, China) and stored at −80 °C. The quality and integrity of the total RNAs were evaluated using Nanodrop 2000 (Thermo Scientific, Wilmington, DE, USA) and Bioanalyzer Chip RNA 7500 series II (Agilent, Santa Clara, CA, USA) instruments.

### Identification of HbACBP family genes

Genes encoding protein HbACBP sequences that possess one ACB domain were identified using the conserved *A. thaliana* ACB domains as a query to search the CDS sequences from the *H. brasiliensis* genome database [23]. The matched sequences with one ACB domain were further analyzed for conserved domains using the motif database Pfam (http://pfam.sanger.ac.uk/) or the Pfam27.0 web server (http://pfam.xfam.org/) [26]. The cDNA sequences of the *HbACBP* (*HbACBP1-HbACBP6*) genes were confirmed by nucleotide re-sequencing and have been deposited in the NCBI database with accession numbers ranging from KY934263–934268.

### Sequence and phylogenetic analysis

The deduced amino acid sequences of the HbACBP proteins, together with those of the *A. thaliana* ACBPs, were aligned using the log-expectation (MUSCLE) alignment tool (http://www.ebi.ac.uk/Tools/msa/muscle) with the default program options [27]. The phylogenetic tree was inferred by the Maximum Likelihood method and 100 bootstrap replicates were employed in each analysis to maximize the statistical significance [8]. Then the tree was visualized by MEGA5.05 software [28].

### Transcript abundance analysis by RT-qPCR

The first-strand cDNAs were synthesized using a PrimerScript RT reagent kit with a gDNA eraser (Takara, Dalian, China). The real-time quantitative PCR (RT-qPCR) analysis was carried out using a SYBR Premix Ex Taq ™ II kit (Takara) on a CFX96 Real-Time System (Bio-Rad, Hercules, CA, USA). The PCR reactions were performed using a 20 μL volume sample with the following parameters: 30 s at 95 °C for pre-denaturation, followed by 40 cycles of 95 °C for 10 s, 58 °C for 20 s and 72 °C for 30 s. The relative expression abundance was determined using the △△Cq method found in the Bio-Rad CFX Manager 3.0 program (Bio-Rad), or by the 2^-△△CT^ method [29], and was calculated from three independent experiments with *H. brasiliensis 18S rRNA* gene as an internal reference. Specific primers for all the selected genes and their efficiencies are listed in the Supporting Information (see Additional file 1). The statistical significance of the different relative transcript abundances was analyzed using SPSS software version 19.0 (Chicago, IL, U.S.A.), and one-way ANOVA with the Student-Newman-Keuls test was used for multiple comparison test to investigate significant differences between groups at the *p* < 0.05 and <0.01 levels.

### Subcellular localization analysis

The subcellular locations of HbACBP1 and HbACBP2 were determined. The complete HbACBP1 (276 bp) and HbACBP2 (855 bp) coding sequences were respectively fused in-frame with green fluorescent protein (GFP) and ligated into the pBWA(V)HS vector (See Additional files 2 and 3) to generate HbACBP1-GFP and HbACBP2-GFP fusions under the control of the CaMV 35S promoter. Their specific primer pairs are listed in Supporting Information (see Additional file 1). The constructs in the destination vectors were confirmed by nucleotide sequencing to verify that the correct fragments were cloned in frame.

Vectors encoding fluorescent protein fusions were individually transfected or co-transfected with pBWA(V)HS-BIP2-mKate, where BIP2 is an ER marker [30], into the mesophyll protoplast cells prepared from the leaves of *A. thaliana* seedlings cultivated at 25 °C for 10 days using the polyethylene glycol method [31]. The transformed protoplasts were examined under a laser-scanning confocal microscope (Olympus FV1000, Tokyo, Japan).

## Results and discussion

### The *H. brasiliensis* ACBP family

**Fig. 1:**
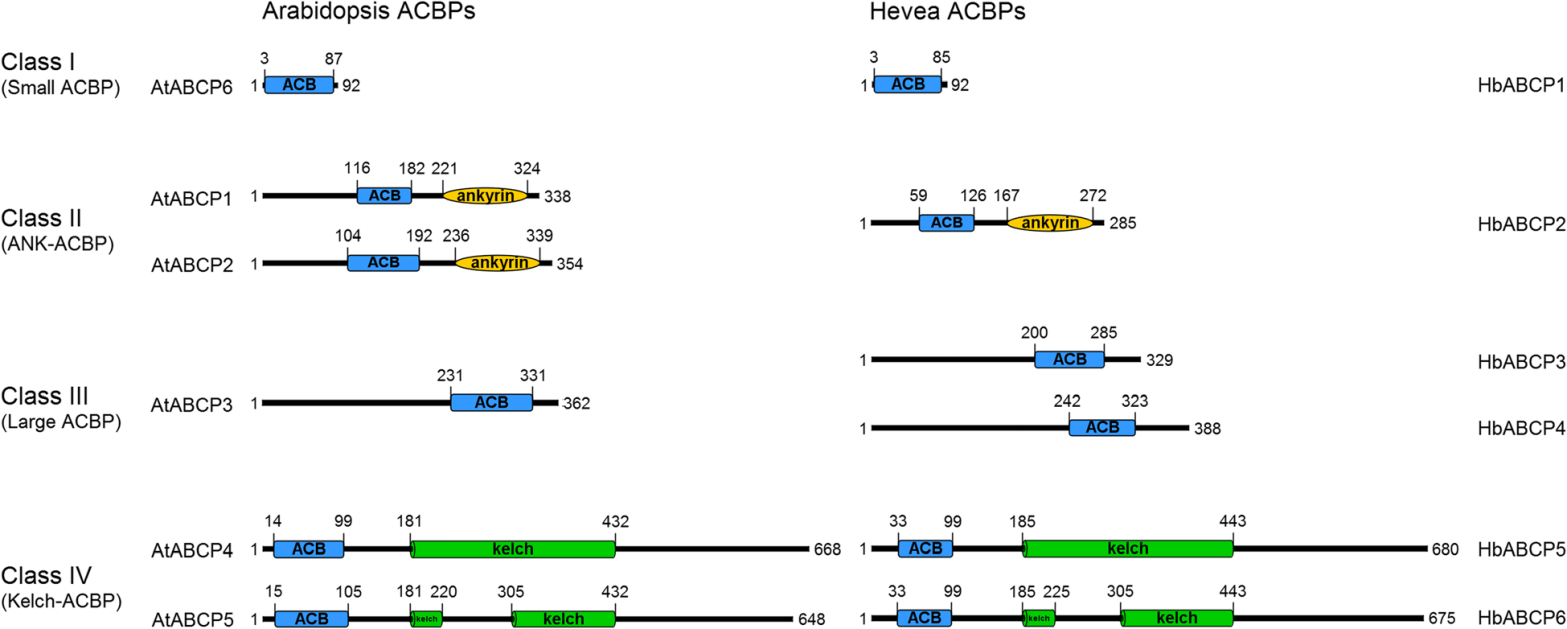
Schematic domain structures of the *Arabidopsis* and *H. brasiliensis* ACBPs. *Arabidopsis* and *H. brasiliensis* ACBPs are designated as AtACBP1-AtACBP6 and HbACBP1-HbACBP6, respectively. The ACB domain, ankyrin repeats, and kelch motifs are labeled in blue, yellow, and green

A total of six ACBP family genes were identified in the *H. brasiliensis* genome, and were designated as *HbACBP1*-*HbACBP6*, which were similar to those of the model plants *A. thaliana* and rice. After mapping the cDNA sequences of the HbACBP genes to the *H. brasiliensis* genome, the exons and introns for each HbACBP gene were predicted and are shown in Additional file 4. The domain structures of the ACBPs from *A. thaliana*, *O. sativa* ACBPs, and other plant species [8, 11] can be used to divide the six *H. brasiliensis* ACBPs into four classes (Fig. 1). Class I *H. brasiliensis* ACBP contained only one member, HbACBP1 with 92 amino acids, which is similar to *A. thaliana* ACBP6 (AtACBP6), and class II also had only one member, i.e., HbACBP2 with 285 amino acids that consist of an ACB domain and ankyrin repeats. HbACBP3 and HbACBP4 were in Class III, which is a large ACBP class that contains only one ACB domain like the small HbACBP1 in Class I. HbACBP5 and HbACBP6 belonged to class IV, which possesses an ACB domain and a kelch motif, and they shared a very high identity and had a very similar genomic structure to each other as shown in Additional file 4.

**Fig. 2:**
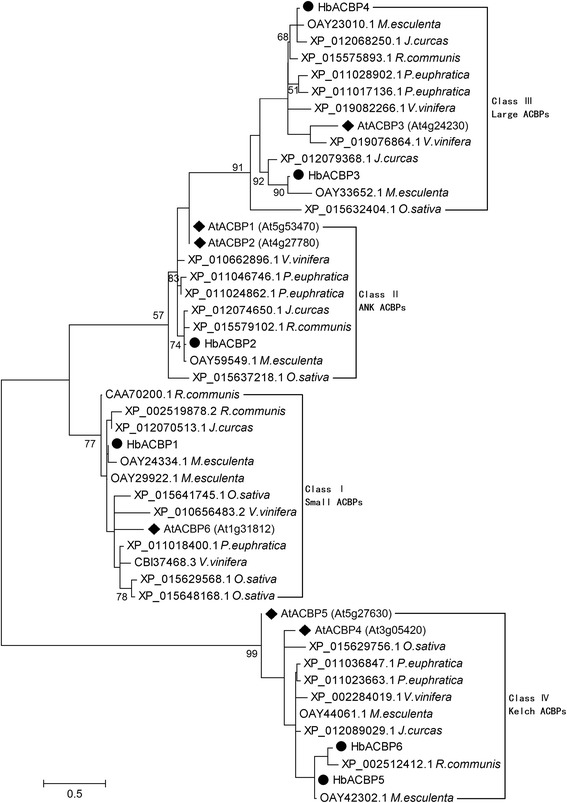
Phylogenetic tree of the ACBPs from *H. brasiliensis* and other plant species. The amino acid sequences of all the selected proteins were aligned using the MUSCLE program and subjected to phylogenetic analysis by the Distance with Maximum Likelihood method using MEGA5.05 software. The numbers on the nodes indicate the bootstrap values (> 50%) after 100 replicates. The scale bar indicates the estimated number of amino acid substitutions per site. ● indicates the six rubber tree ACBPs;♦ indicates *Arabidopsis* ACBPs

*H. brasiliensis* belongs to the spurge (Euphorbiaceae) family, which includes other economically important crops, such as cassava (*Manihot esculenta*), castor bean (*Ricinus communis*), and physic nut (*Jatropha curcas*) etc. The genome DNAs of the four crop species have been sequenced [23, 32–34], which allows the gene functions among different species in the spurge family to be compared. After searching the conserved ACB domains, seven, five, and five ACBP members were identified in the *M. esculenta*, *R. communis*, and *J. curcas* genomes, respectively. A phylogenetic tree analysis demonstrated that every rubber tree ACBP was clustered with those of other closely related species, such as *M. esculenta*, *R. communis*, and *J. curcas*, but were further from those of *A. thaliana*, a distantly related species (Fig. 2). These results further confirm that there is a high conservation and evolutionary relationship between ACBPs in land plants.

### Organ/Tissue-specific expression patterns of the HbACBP genes

**Fig. 3:**
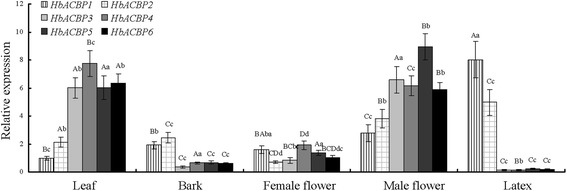
Expression patterns of the HbACBP genes in five different rubber tree organs or tissues. Three independent samples were used for RT-qPCR analysis, and the transcript levels of each gene were determined. The values are shown as the mean ± S. D of three independent biological replicates. Different uppercase and lowercase letters above the bars indicate differences between mean values within each gene at the 0.01 and 0.05 significance levels, respectively

The expression patterns of the HbACBP genes were determined in five rubber tree organs or tissues using RT-qPCR. The expressions of six HbACBP transcripts were all detected in mature leaves, bark tissues, female flowers, male flowers, and latex, but the patterns were very different. *HbACBP1* and *HbACBP2* were more predominantly expressed in the latex compared to *HbACBP3*-*HbACBP6*, and were more highly expressed in the latex than in other organs or tissues. *HbACBP3*-*HbACBP6* had almost the same expression profiles with relatively high expressions in mature leaf and male flower, moderate expressions in female flower and bark tissue, and markedly low expression in latex (Fig. 3). The results indicate that *HbACBP3-HbACBP6* might play more important roles in the development of the *H. brasiliensis* leaf and the male flower but not in the latex production of rubber trees.

Latex is collected through regular bark tapping and is actually the cytoplasm of the laticifer cells in rubber trees [35]. It is the main commercial source of high-quality natural rubber worldwide [36]. Anatomical studies have demonstrated that the *H. brasiliensis* laticifer has no plasmodesmata and is apoplastically isolated from adjacent cells in the inner bark [22], which might explain why *HbACBP3* and *HbACBP4* were barely expressed in the laticifers (Fig. 3), since HbACBP3 and HbACBP4 belong to class III ACBPs that are the extracellularly-targeted proteins [17].

### Expression profiles of the *HbACBPs* in the laticifers of rubber trees

**Fig. 4:**
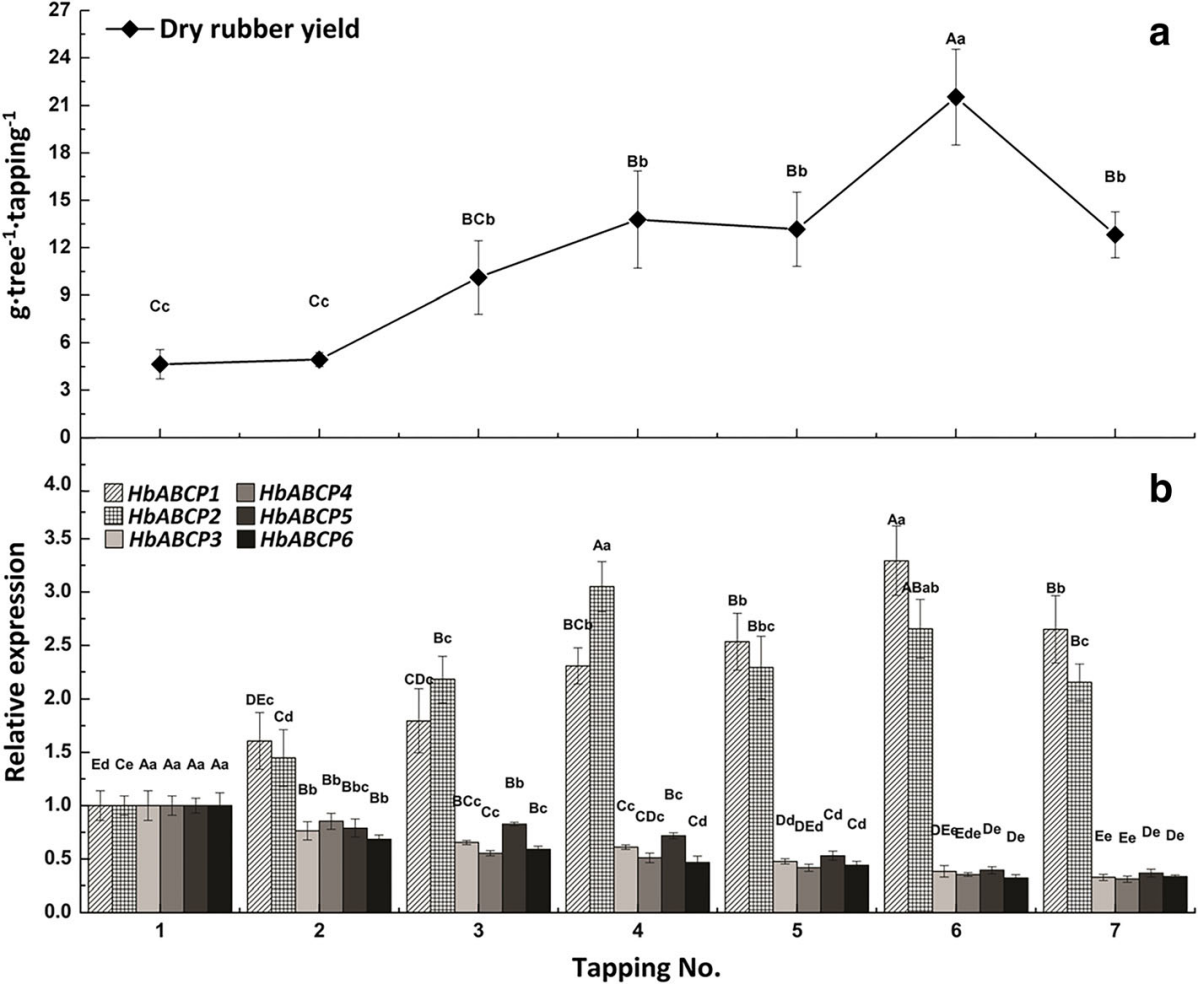
Effect of consecutive bark tappings on dry rubber yield and the HbACBP genes expression. **a** Effect of consecutive bark tapping on dry rubber yield, **b** Effects of bark tapping on the expression of HbACBP1 and HbACBP2 genes in the latex of rubber trees. Fresh latex was collected from mature and virgin rubber trees from every bark tapping. The transcript abundance of each gene was detected by RT-qPCR. The standard bars were obtained from at least three independent replications and the results were given as mean ± S.D. Different uppercase and lowercase letters above the bars indicate differences between mean values within each gene at the 0.01 and 0.05 significance levels, respectively

**Fig. 5:**
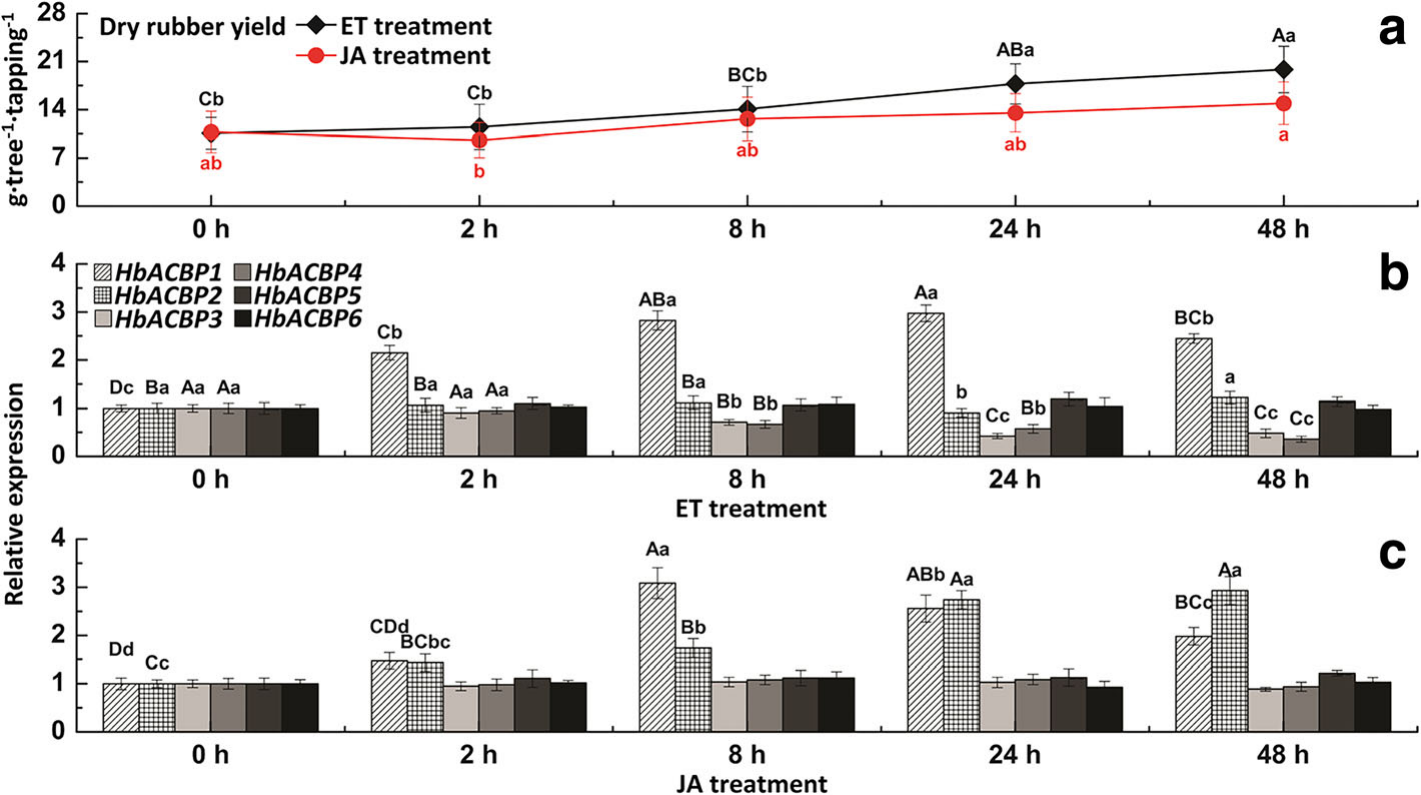
Comparison of ET and JA stimulation on dry rubber yield and HbACBP genes expression. **a** Changes in dry rubber yield, **b** Expression patterns of HbACBP genes in the latex of rubber trees subjected to ET treatment, **c** Expression patterns of HbACBP genes in the latex of rubber trees subjected to JA treatment. Fresh latex was collected from exogenous ET- or JA-treated rubber trees that were regularly tapped for 2 years. The transcript abundance of each gene was detected by RT-qPCR. The standard bars were obtained from at least three independent replications and the results were given as mean ± S.D. Different uppercase and lowercase letters above the bars indicate differences between mean values within each gene at the 0.01 and 0.05 significance levels, respectively

**Fig. 6:**
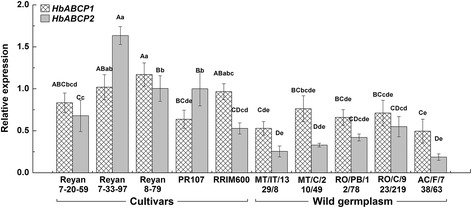
Differential expressions of the HbACBP1 and 2 genes in different rubber tree clones or germplasms. Fresh latex was collected from 5 Amazon wild germplasms and 5 cultivars rubber tree clones. The transcript abundance of each gene was detected by RT-qPCR. The standard bars were obtained from at least three independent replications and the results were given as mean ± S.D. Different uppercase and lowercase letters above the bars indicate differences between mean values within each gene at the 0.01 and 0.05 significance levels, respectively

In rubber trees, the most important agronomic trait is the latex yield, which is highly related to latex metabolism and regeneration in the laticifers. Many studies have investigated the molecular events occurring in the laticifers of rubber trees using transcriptome or proteome techniques [37–40]. Herein, the expression profiles of the six HbABCP genes in the latex were investigated under bark tapping (a mechanical wound), exogenous ET (a releaser of ethylene), and JA stimulation. A RT-qPCR analysis showed that the HbACBP genes had diverse expression patterns in response to the treatments, which is consistent with the inducible expression of the ACBP genes. Bark tapping can significantly increase the transcript levels of *HbACBP1* and *HbACBP2*, but greatly suppress the expression of *HbACBP3*-*HbACBP6* (Fig. 4). In comparison with bark tapping, ET can greatly enhance the expression level of *HbACBP1* (Fig. 5), and slightly increase *HbACBP5* expression although this result was not significant (fold change ≤1.5), but had almost no effects on the expression of *HbACBP2* and *HbACBP6*, and greatly suppressed the expression of *HbACBP3* and *HbACBP4* (Fig. 5). JA can significantly induce the expression of *HbACBP1* and *HbACBP2*, but had no influence on other HbACBP genes (Fig. 5). These data indicated *HbACBP1* and *HbACBP2* are the two major ACBP genes that are predominantly expressed in the laticifers and can be induced by bark tapping and treatments with ET and JA. In addition, the different expression patterns of *HbACBP1* and *HbABCP2* between 5 rubber tree cultivars and 5 Amazon wild germplasms indicate that *HbACBP1* and *HbABCP2* had higher expression levels in the laticifers of the rubber tree cultivars with higher latex production than the Amazon wild germplasms with relatively lower latex yield (Fig. 6).

The characteristic gene expression of *HbACBPs* in the latex revealed that *HbACBP1* and *HbACBP2* play pivotal roles during lipid metabolism and responses to bark tapping or ET, JA stimulation, and that they are significantly related to latex metabolism and production in rubber trees. ET (applied as chloro-2-ethylphosphonic acid) is commonly used to stimulate latex production [41]. JA is a key inducer of laticifer differentiation [42], and might be a regulator of rubber biosynthesis-related genes [43] because it acts as an elicitor during the induction of plant secondary metabolites [44]. Bark tapping might trigger the latex metabolic switch to turn on, and can activate latex regeneration and rubber biosynthesis, which enhances latex yield [45]. Therefore, further investigation is needed to elucidate HbACBP1- and HbACBP2-mediated lipid metabolism and phospholipidic signaling, and their relationships with latex metabolism in the *H. brasiliensis* laticifers.

### Subcellular localization and possible functions of HbACBP1 and HbACBP2

**Fig. 7:**
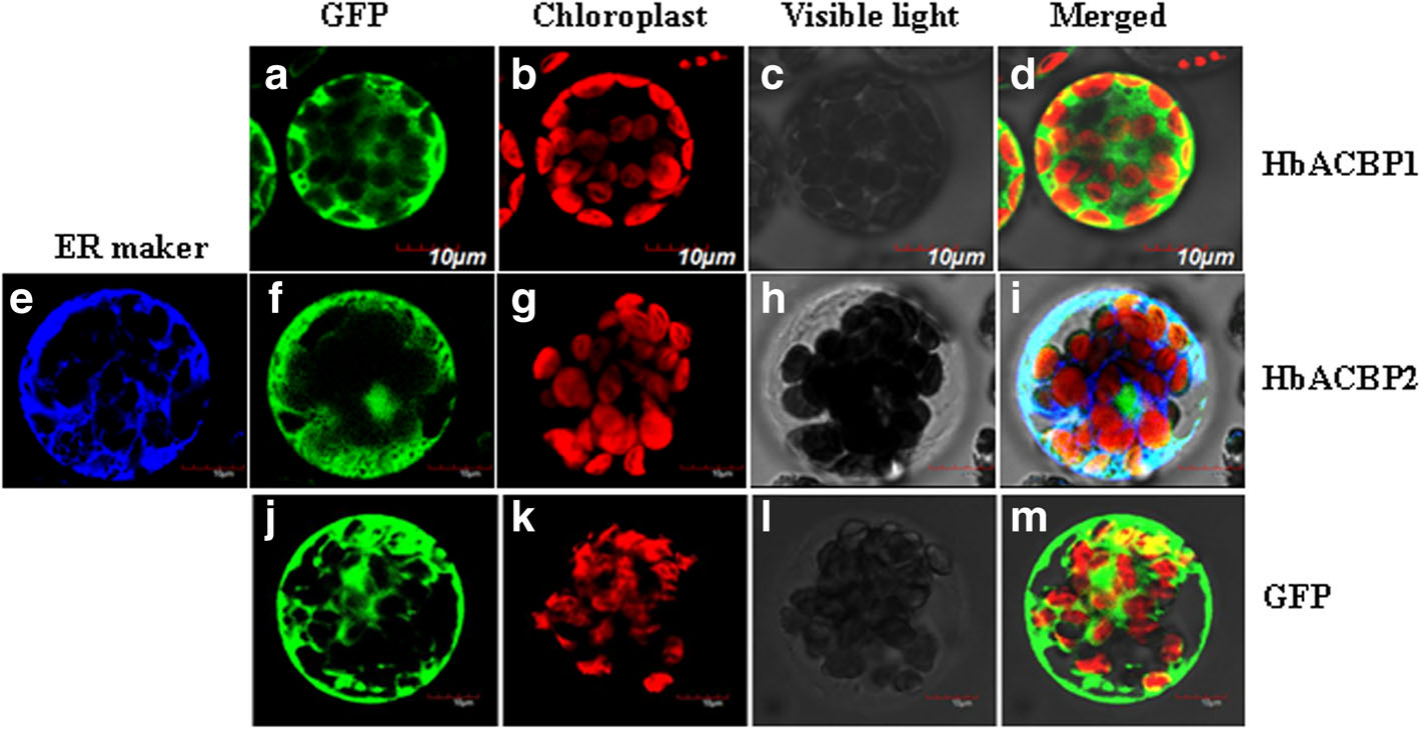
Subcellular localization of HbACBP1 and HbACBP2 in *Arabidopsis* leaf protoplasts. **a**-**d** HbACBP1-GFP fusion protein; **e**-**i** co-transfection of HbACBP2-GFP fusion protein and the ER marker; **j**-**m** GFP control; **a**, **f**, and **j** transient expression of GFP; **b**, **g**, and **k** chloroplast autofluorescence; **c**, **h**, and **l** visible light; **d**, **i**, and **m**, merged images; and **e** ER marker. Scale bar = 10 μm

The biological function of a protein is closely related to its subcellular localization. To investigate the possible involvement of HbACBPs in latex metabolism, the predominantly laticifer-expressed HbACBP1 and HbACBP2 were further selected for subcellular localization analysis. HbACBP1 and HbACBP2 were fused with the GFP, and were transiently expressed in *A. thaliana* leaf protoplasts. ACBP6 (AtACBP6), a class I ACBP, is localized in the cytosol [7], which is the same as *A. thaliana.* HbACBP1 is also a cytosol protein (Fig. 7 a-d). HbACBP2-GFP was co-transfected into *A. thaliana* leaf protoplasts with mKate-tagged ER marker BIP2, and the results showed that HbACBP2 is an ER-associated protein but is not in the PM (Fig. 7 e-i), which agrees with results that show that AtACBP1/2 is predominantly located in the ER [7].

A recent study reported that AtACBP6 is involved in JA-biosynthesis of the sieve elements in *A. thaliana* phloem tissues [21]. Therefore, an intriguing suggestion is that HbACBP1 is potentially involved in JA formation in the laticifer cells of rubber trees, since JA acts as a key regulator during laticifer differentiation and rubber biosynthesis [42, 43]. In rubber tree phloem, both laticifer cells and sieve elements originate from the vascular cambia, but they belong to two different kinds of cell types and do not have any linkages (including plasmodesmata) between them [46]. As far as the above-mentioned studies are concerned, a JA-biosynthesis pathway, which is independent from that in the sieve elements and other cells, might exist in the laticifer cells, and this hypothesis was supported by the identification of all the genes related to JA-biosynthesis pathway in the isolated latex of the laticifers.

In *A. thaliana*, AtACBP6 may regulate JA composition by affecting the function of CTS [21]. CTS is an ATP-binding cassette (ABC) transporter (At4g39850, AtABCD1) belonging to subfamily ABCD, and is used to transport JA precursors into peroxisomes [47]. The rubber tree latex transcriptome analysis identified two ABCD genes, *HbABCD1* and *HbABCD2* [24]. Both HbACBD1 and AtABCD1 comprise 1337 amino acids with a (TMD-NBD)_2_ domain structure. They have an identity of 78% for their amino acid sequences, which implies that HbACBD1 may have the same role as the *A. thaliana* CTS (AtABCD1), and the functional relationship between HbACBP1 and HbACBD1 needs to be further revealed during JA-biosynthesis in rubber tree laticifers. Rubber trees often suffer from regular bark tapping for latex collection. Bark tapping is a mechanical wound and could act as a stress elicitor [48], which can greatly increase the metabolic activity of the laticifers [45]. Since lipid signaling functions importantly in plant responses to various abiotic stresses [49]; therefore, the integration of lipid signaling and wound signaling into phytohormone-regulated pathways could be used to elucidate the mechanisms underlying the latex metabolism of rubber trees.

## Conclusions

Six members of the *H. brasiliensis* ACBP family were identified and structurally compared to *A. thaliana* ACBPs. The HbACBP genes are organ/tissue-specifically expressed and have different expression patterns in response to stimulation by ET, JA, and bark tapping (mechanical wound). *HbACBP1* and *HbACBP2* are more prominently expressed in the *H. brasiliensis* laticifers. Their expressions can be markedly increased by bark tapping or ET, JA stimulation, and have significant correlation with latex production, which suggest that HbACBP1 and HbACBP2 play crucial roles in the lipid and latex metabolism in rubber tree laticifers. Further investigations are needed to elucidate the relationship between the ACBP-mediated lipid metabolism and latex production of rubber trees.

## Additional files

Additional file 1: Primers and their sequences used in this study. (DOC 44 kb)

Additional file 2: The vector of HbACBP1 for subcellular localization. (TIFF 911 kb)

Additional file 3: The vector of HbACBP2 for subcellular localization. (TIFF 914 kb)

Additional file 4: Linear presentation of the exon-intron structures for the *Hevea* ACBP family genes. (TIFF 430 kb)

## Abbreviations

*A. thaliana*: *Arabidopsis thaliana*
ACBD: acyl-CoA-binding domain
ACBP: Acyl-CoA-binding protein
CTS: COMATOSE
ER: Endoplasmic reticulum
ET: Ethephon
GFP: Green fluorescent protein
*H. brasiliensis*: *Hevea brasiliensis*
JA: Jasmonic acid
PM: Plasma membrane
RT-qPCR: Real-time quantitative PCR
