# Supplementary material for: Acyl-CoA-binding protein family members in laticifers are possibly involved in lipid and latex metabolism of Hevea brasiliensis (the Para rubber tree)
Source: BMC Genomics. 2018 Jan 2;19:5. doi: 10.1186/s12864-017-4419-6 (PMC5751871; doi:10.1186/s12864-017-4419-6)
Supplement: Supplementary file 1 — Primers and their sequences used in this study. (DOC 44 kb) [file 12864_2017_4419_MOESM1_ESM.doc]

**Additional file 1**

**Table S1**. Primers and their sequences used in this study

| Primer names | Primer sequences（5’-3’） | Annotation |
| --- | --- | --- |
| *Hb18S rRNA* F | GCTCGAAGACGATCAGATAC | Reference gene for real-time RT-PCR |
| *Hb18S rRNA* R | TTCAGCCTTGCGACCATAC |
| *HbACBP1* F | TTGAAGGAGGAATTTGAGG | Real-time RT-PCR |
| *HbACBP1* R | CCAACAGTGGCTTGCTTAT |
| *HbACBP2* F | GAGGAGGAATCTGGAAATG | Real-time RT-PCR |
| *HbACBP2* R | GTCCGACCCTCACTATCTT |
| *HbACBP3* F | GAACATGAGCCCTGAGGTG | Real-time RT-PCR |
| *HbACBP3* R | TTGTTGCGTCATTGTCTGC |
| *HbACBP4* F | GTATCGTATAAAGTACCGGGTTG | Real-time RT-PCR |
| *HbACBP4* R | TTTGGATGATGGGATGGAG |
| *HbACBP5* F | TGCTACAAATGCTACCAGAG | Real-time RT-PCR |
| *HbACBP5* R | CCAGTTCTTCTTTTTCCGAC |
| *HbACBP6* F | GCTACCAGAGATTTGGAATTG | Real-time RT-PCR |
| *HbACBP6* R | CCAGTTCTTCTTTTTCTGCCT |
| *HbACBP1* F | *GGTCTCacaacatgggtttgaaggaggaatt | ORF cloning for subcellular location |
| *HbACBP1* R | *GGTCTCatacaagcagaagcagcagcagcag |
| *HbACBP2* F | *GGTCTCacaacatggctgagcacggtagcgt | ORF cloning for subcellular location |
| *HbACBP2* R | *GGTCTCatacatgcccgcaggcaaggccagt |

*GGTCTC is the restriction enzyme recognizing site of the BsaI/Eco31I.
