# Supplementary figures and images for: Acyl-CoA-binding protein family members in laticifers are possibly involved in lipid and latex metabolism of Hevea brasiliensis (the Para rubber tree)
Source: BMC Genomics. 2018 Jan 2;19:5. doi: 10.1186/s12864-017-4419-6 (PMC5751871; doi:10.1186/s12864-017-4419-6)

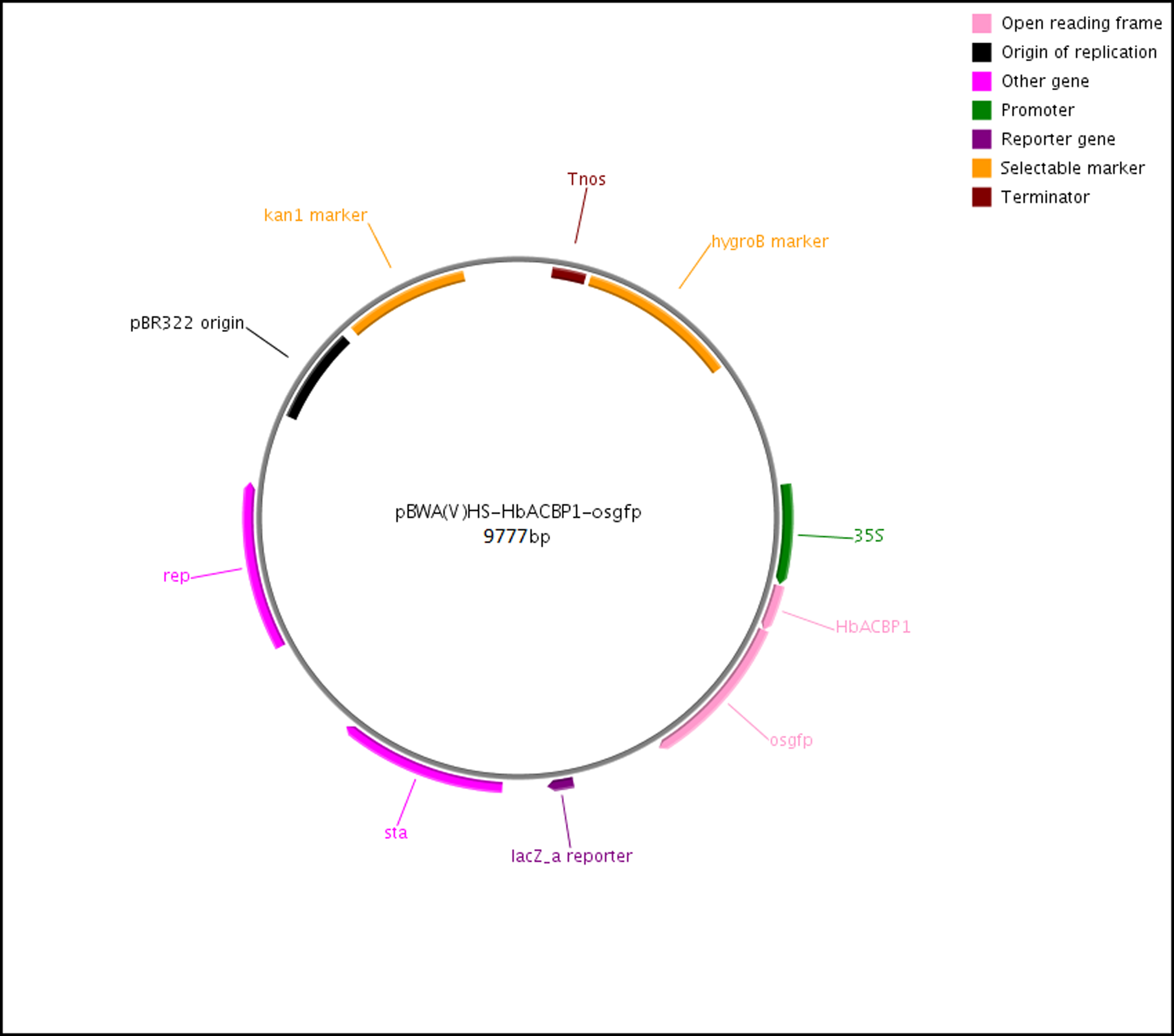

Supplement: Supplementary file 2 — The vector of HbACBP1 for subcellular localization. (TIFF 911 kb) [file 12864_2017_4419_MOESM2_ESM.tif]

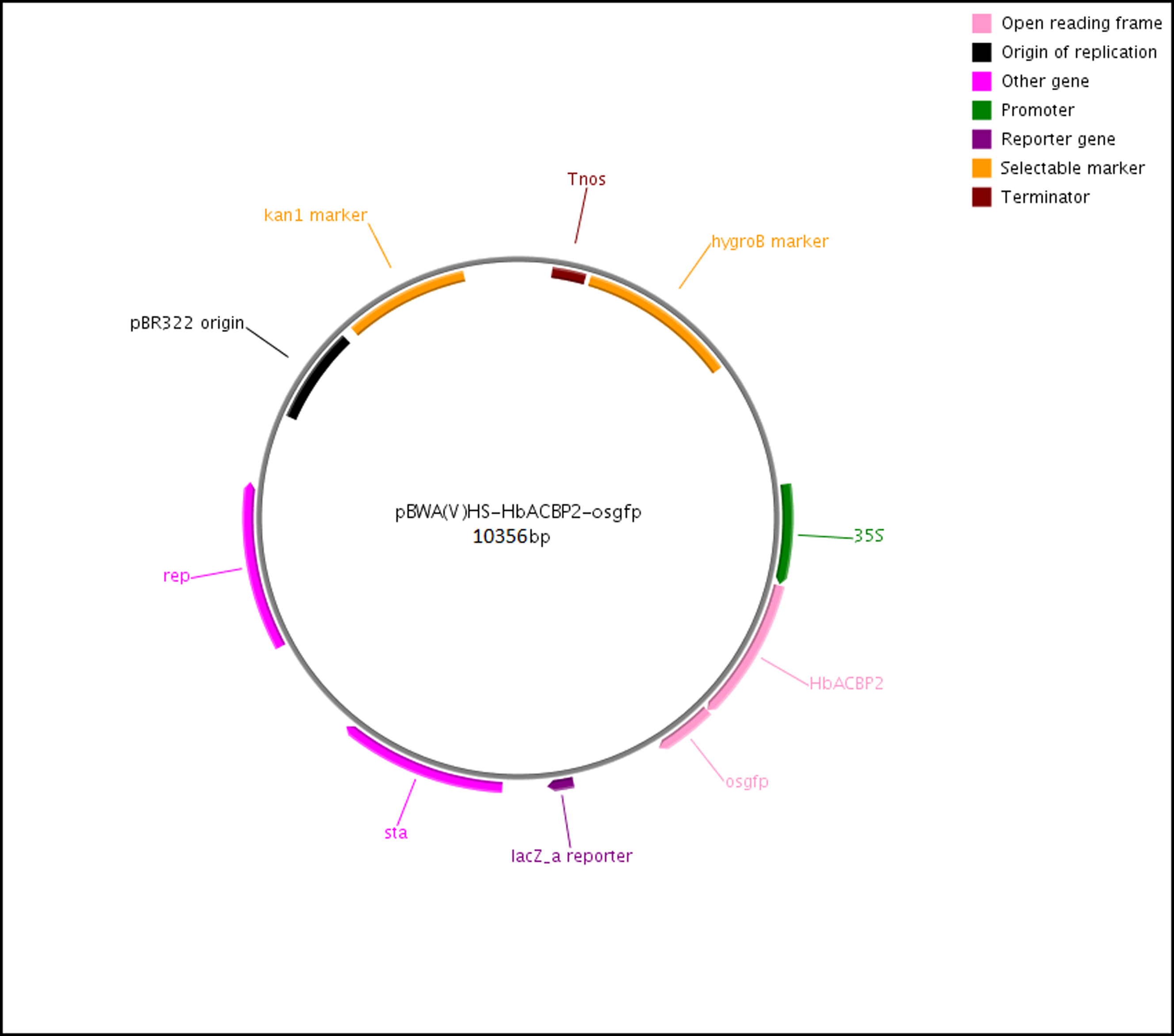

Supplement: Supplementary file 3 — The vector of HbACBP2 for subcellular localization. (TIFF 914 kb) [file 12864_2017_4419_MOESM3_ESM.tif]

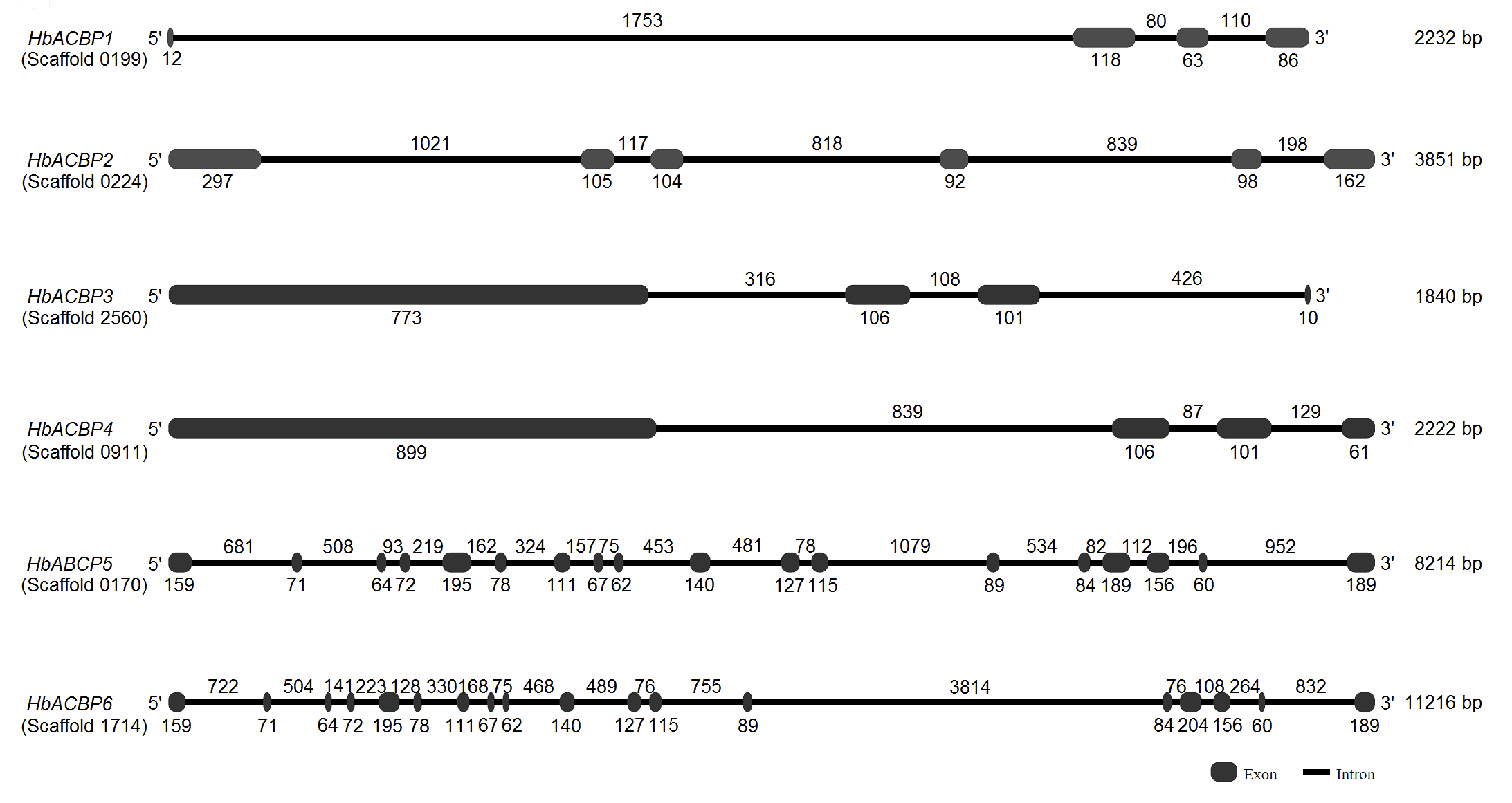

Supplement: Supplementary file 4 — Linear presentation of the exon-intron structures for the Hevea ACBP family genes. (TIFF 430 kb) [file 12864_2017_4419_MOESM4_ESM.tif]
